# The Adverse Effects of Triptolide on the Reproductive System of *Caenorhabditis elegans*: Oogenesis Impairment and Decreased Oocyte Quality

**DOI:** 10.3390/ijms18020464

**Published:** 2017-02-21

**Authors:** Qinli Ruan, Yun Xu, Rui Xu, Jiaying Wang, Yongqing Hua, Meng Wang, Jinao Duan

**Affiliations:** 1 Center for Drug Safety Evaluation and Research, Nanjing University of Chinese Medicine, Nanjing 210023, China; nzyxuyun@gmail.com (Y.X.); xurui@njucm.edu.cn (R.X.); wangjy@njucm.edu.cn (J.W.); wangm@njucm.edu.cn (M.W.); 2 Jiangsu Collaborative Innovation Center of Chinese Medicinal Resources Industrialization, Nanjing University of Chinese Medicine, Nanjing 210023, China; hua_yq@njucm.edu.cn (Y.H.); dja@njucm.edu.cn (J.D.); 3 Jiangsu Key Laboratory for High Technology Research of Traditional Chinese Medicine Formulae, Nanjing University of Chinese Medicine, Nanjing 210023, China

**Keywords:** *Caenorhabditis elegans*, Triptolide, oogenesis, mitosis, apoptosis

## Abstract

Previous studies have revealed that Triptolide damages female reproductive capacity, but the mechanism is unclear. In this study, we used *Caenorhabditis elegans* to investigate the effects of Triptolide on the germline and explore its possible mechanisms. Our data show that exposure for 4 h to 50 and 100 mg/L Triptolide reduced *C. elegans* fertility, led to depletion and inactivation of spermatids with the changes in the expression levels of related genes, and increased the number of unfertilized oocytes through damaging chromosomes and DNA damage repair mechanisms. After 24 and 48 h of the 4 h exposure to 50 and 100 mg/L Triptolide, we observed shrink in distal tip cells, an increase in the number of apoptotic cells, a decrease in the number of mitotic germ cells and oocytes in diakinesis stage, and chromatin aggregates in −1 oocytes. Moreover, expression patterns of the genes associated with mitotic germ cell proliferation, apoptosis, and oocyte quality were altered after Triptolide exposure. Therefore, Triptolide may damage fertility of nematodes by hampering the development of oocytes at different developmental stages. Alterations in the expression patterns of genes involved in oocyte development may explain the corresponding changes in oocyte development in nematodes exposed to Triptolide.

## 1. Introduction

Triptolide is the most active diterpenoid triepoxide chemical among the ingredients isolated from the root of *Tripterygium wilfordii*. Pharmacological studies have shown that Triptolide has various pharmacological properties, including anti-inflammatory and anti-tumor effects, and it also affects immune regulation [1,2,3,4,5,6]. Because of these properties, Triptolide has been used to treat various diseases (e.g., rheumatism, asthma, autoimmune diseases, diseases of kidney and the skin, etc.) and tumors, and has also been used in organ transplantation [7,8].

Unfortunately, the use of Triptolide has been limited in the clinical practice due to its side-effects, especially its toxic effects on the reproductive system. A clinical study showed that clinical manifestations of Triptolide include decreased sperm or azoospermia in males, and decreased menstrual quantity or amenorrhoea in females [9]. Previous studies using animal models paid more attention to the adverse effects of Triptolide on the male reproductive system, and less attention to its adverse effects on the female reproductive system. Liu et al. [10] showed that Triptolide doses of 200 and 400 µg/kg significantly reduced the relative weights of the ovaries and the uterus of female Sprague Dawley (SD) rats. Using a qualitative histological analysis of the ovaries, this study revealed a reduction in developing follicles and an increase in atretic follicles in SD rats treated with Triptolide. This observation suggested that Triptolide had a direct effect on the ovaries and inhibited the development of oocytes. However, the possible mechanisms responsible for these effects need to be further explored.

*Caenorhabditis elegans*, a thoroughly studied animal model, is considered a useful alternative for mammalian toxicity assays [11]. Because of *C. elegans* short lifecycle, cellular simplicity, easy maintenance, sublethal endpoints and convenient observation methods, this nematode has been used for basic toxicity assessments. Additional features making *C. elegans* suitable for mechanistic studies in environmental and biomedical toxicology include a clear genetic background, genetic manipulability, and evolutionarily-conserved signaling pathways.

*C. elegans* has a simple but highly differentiated reproductive system with a complete process of gametogenesis [12]. At the distal end of each hermaphrodite gonad arm is the somatic distal tip cell (DTC). *C. elegans* germline displays a distal-to-proximal polarity proliferation, meiotic prophase progression, and oogenesis. Comparative biology studies have shown that the oogenesis process is very similar in nematodes and mammals [13,14]. In addition, comparative genomics studies have shown that signaling pathways regulating apoptosis in *C. elegans* are highly conserved, relative to the mammalian counterparts [15,16]. In recent years, *C. elegans* has been used to investigate the adverse effects of chemicals, such as arsenic, pesticide chlorpyrifos and endosulfan isomers, and environmental pollutants, on the reproductive system [17,18,19,20,21]. In these studies, germ cell apoptosis and mitotic germ cell proliferation have been intensively investigated, however, oocyte in diakinesis has been seldom studied.

In this study, we employed *C. elegans* to perform a reproductive toxicity assessment of Triptolide in the perspective of fairly systematic oogenesis. We sought to explore the possible underlying mechanisms of Triptolide-induced reproductive toxicity affecting the oogenesis of nematodes. These data are helpful to understanding the possible mechanisms underlying the documented reproductive toxicity of Triptolide in vivo. Our study highlights the usefulness of *C. elegans* for the assessment of the possible adverse effects of drugs on the female reproductive system.

## 2. Results

### 2.1. Effects of Triptolide on Nematodes Viability and Body Length

**Figure 1:**
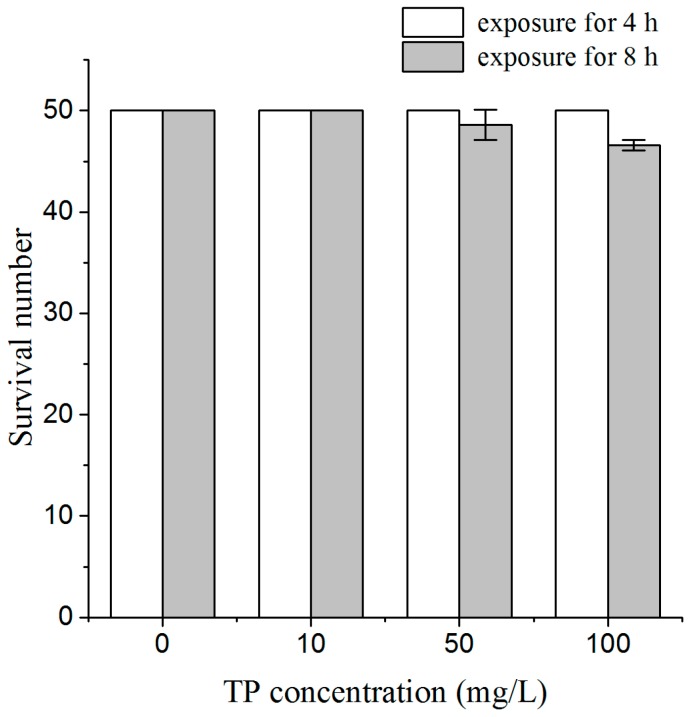
Survival of nematodes exposed to TP for 4 and 8 h. TP, Triptolide. Note: There are no error bars for the control and 10 mg/L Triptolide groups because there were no dead nematodes in the three repeated experiments.

**Figure 2:**
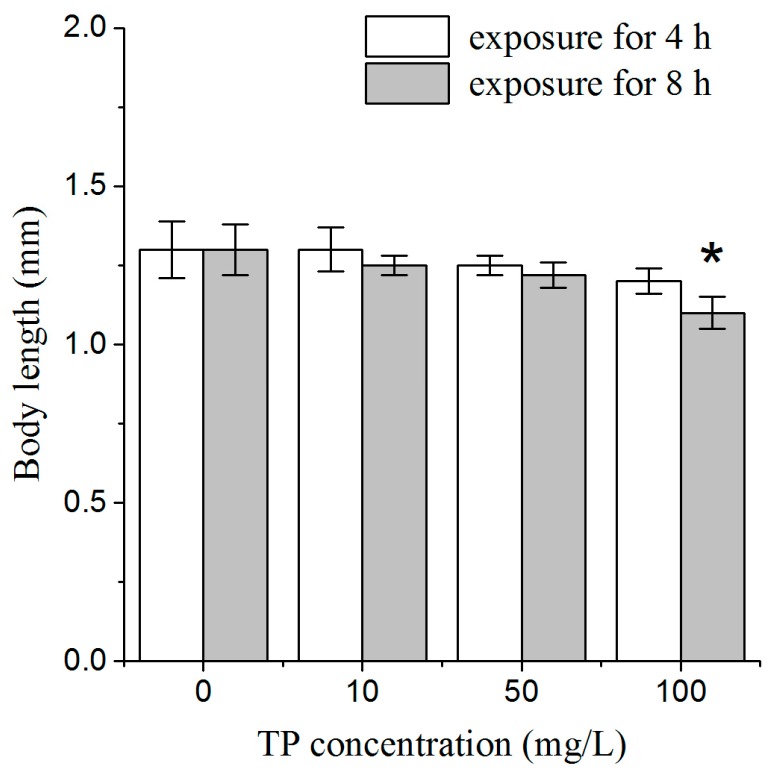
Comparison of body length in nematodes exposed to TP for 4 and 8 h. TP, Triptolide. Bars represent means ± SEM. * *p* < 0.05 vs. the control group.

To assess the whole-body effects of a short time exposure to Triptolide on nematodes, we used lethal toxicity and body length as endpoint measures. After 4 h of exposure to Triptolide, we did not find dead nematodes, and there was no obvious effect on body length, relative to the un-exposed controls. After 8 h of exposure to Triptolide, one nematode was found dead among 50 nematodes exposed to 50 mg/L Triptolide, and three nematodes were found dead among 50 nematodes exposed to 100 mg/L Triptolide (Figure 1). After 8 h of exposure, the body length of nematodes was significantly reduced in Triptolide at 100 mg/L (Figure 2). These results indicate that 4 h exposure to Triptolide is not lethal to the nematodes and does not inhibit the development of nematodes during the exposure. Therefore, Triptolide exposure was conducted for 4 h in the following toxicity assays.

### 2.2. Effects of Triptolide on Nematodes Reproduction

**Figure 3:**
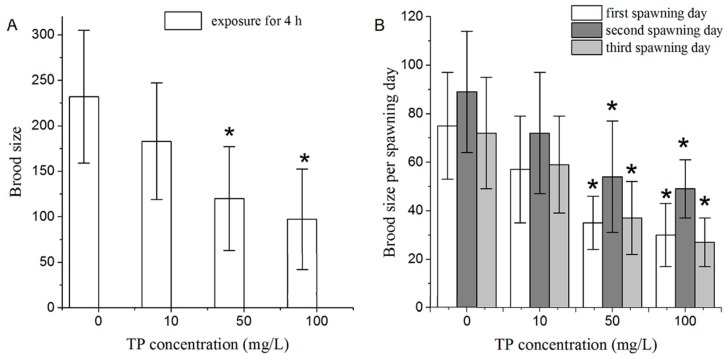
Effects of exposure to TP on the fertility of nematodes: (**A**) effects of 4 h TP exposure on brood size; and (**B**) effects of 4 h TP exposure on brood size in each spawning day. TP, Triptolide. Bars represent means ± SEM. * *p* < 0.05 vs. the control group.

From a toxicological perspective, reproductive organs are one of the most important targets of Triptolide. We investigated the effects of a short time exposure to Triptolide on the reproductive system of *C. elegans* using brood size as an endpoint measure. After 4 h of exposure, Triptolide at 10 mg/L did not have an obvious influence on brood size of nematodes. In contrast, 4 h of exposure to Triptolide at 50 and 100 mg/L significantly reduced brood size of nematodes (Figure 3A).

In terms of brood size in each spawning day, exposure to 50 and 100 mg/L of Triptolide for 4 h significantly reduced brood size laid in the first, second, and third days of the spawning time (Figure 3B). The difference between brood size laid by the control and the Triptolide-exposed (100 mg/L) nematodes was most obvious.

### 2.3. Effects of Triptolide on the Number of Unfertilized Oocytes

**Figure 4:**
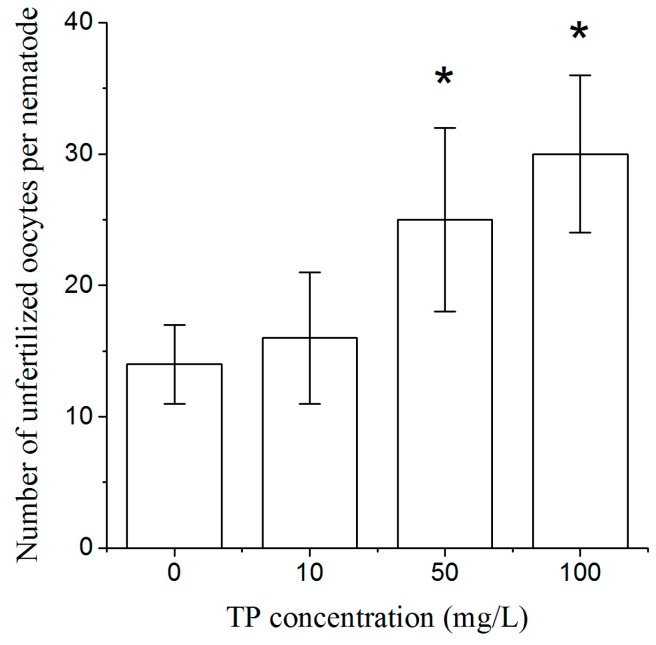
The number of unfertilized oocytes per nematode exposed to TP for 4 h. TP, Triptolide. Bars represent means ± SEM. * *p* < 0.05 vs. the control group.

To explore whether Triptolide affects the production of oocytes, we designed the mating experiments with *him-5* mutant males and exposed hermaphrodites. As shown in Figure 4, exposure to 50 and 100 mg/L Triptolide for 4 h significantly increased the number of unfertilized oocytes per nematode.

### 2.4. Effects of Triptolide on the Number and Morphology of Spermatids

**Figure 5:**
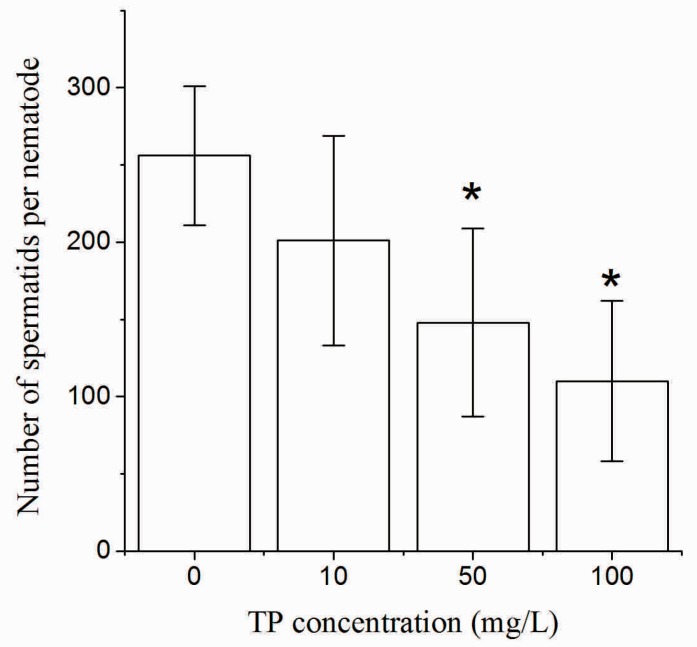
The number of spermatids per nematode exposed to TP for 4 h. TP, Triptolide. Bars represent means ± SEM. * *p* < 0.05 vs. the control group.

After 4 h of exposure to Triptolide, as shown in Figure 5, the number of spermatids of hermaphrodites was significantly decreased in nematodes that were exposed to 50 and 100 mg/L Triptolide.

**Figure 6:**
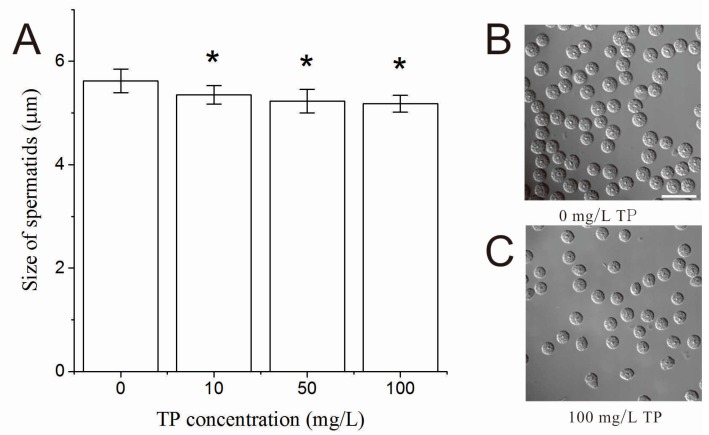
Effects of TP on the size of spermatids: (**A**) the size of spermatids per nematode exposed to TP for 4 h; (**B**) the spermatids morphology of the control group after 6 h of exposure; and (**C**) the spermatids morphology of nematodes exposed to 100 mg/L TP after 6 h of exposure. Scale bar is 15 µm. TP, Triptolide. Bars represent means ± SEM. * *p* < 0.05 vs. the control group.

The size and shape were denoted as the morphology of spermatids of hermaphrodites. After 4 h of exposure to Triptolide, the size of spermatids of hermaphrodites was significantly decreased in nematodes that were exposed to 10, 50 and 100 mg/L Triptolide in a dose-dependent relationship as shown in Figure 6. However, no obvious abnormal spermatids were observed in the exposed nematodes.

### 2.5. Effects of Triptolide on Spermatids Activation

**Figure 7:**
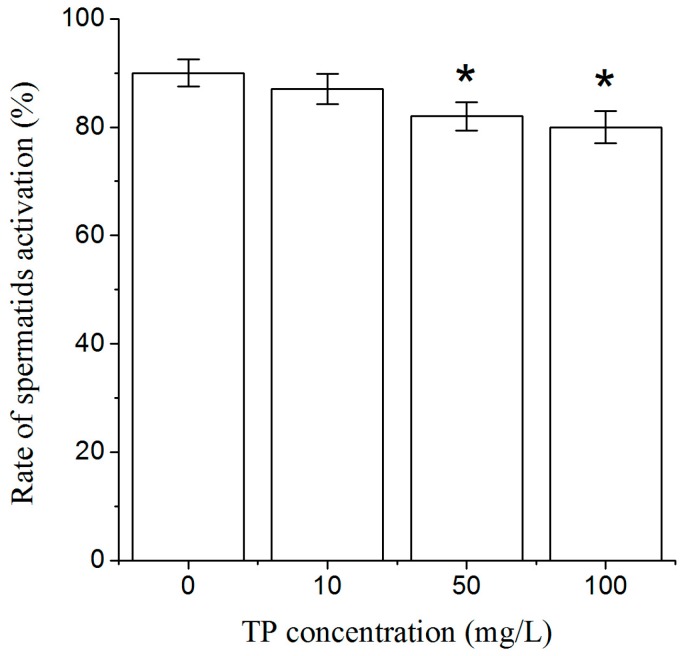
The rate of spermatids activation (%) of nematodes exposed to TP for 4 h. TP, Triptolide. Bars represent means ± SEM. * *p* < 0.05 vs. the control group.

After 4 h of exposure to Triptolide, spermatids were activated by pronase in vitro. The rate of spermatids activation was significantly decreased in nematodes that were exposed to 50 and 100 mg/L Triptolide (Figure 7).

### 2.6. Effects of Triptolide on the Development of DTCs

Gametogenesis occurs during the late L4 and the adult stages of *C. elegans*, and oogenesis occurs during its adult stage. After being exposed to Triptolide for 4 h, *C. elegans* nematodes are still at the stage of young adults, having immature gonads. When the nematodes are mature, they enter into three days of ovulation. Therefore, we selected 24, 48, and 72 h after Triptolide exposure to observe *C. elegans* oocytes at different developmental stages during the entire fertility time.

**Figure 8:**
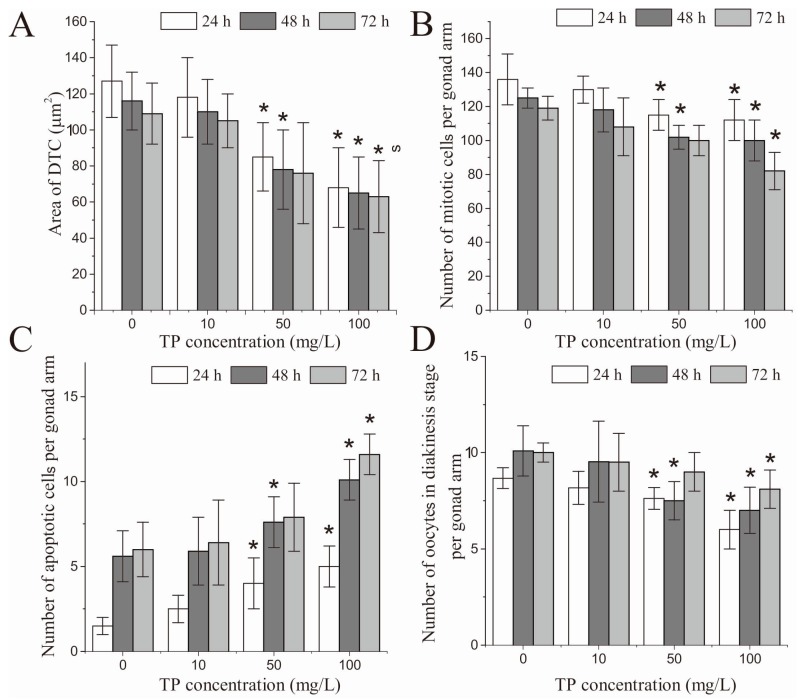

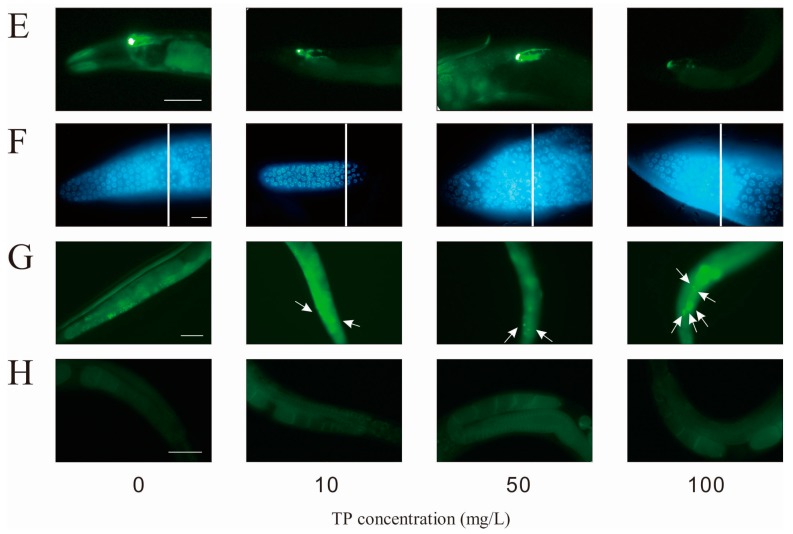
Effects of exposure to TP for 4 h on the oocytes at different developmental stages: (**A**) comparison of the effects of TP on the area of DTCs after 24, 48, and 72 h of exposure; (**B**) comparison of the effects of TP on the number of mitotic germ cells per gonad arm of nematodes after 24, 48, and 72 h of exposure; (**C**) comparison of effects of TP on the number of apoptotic cells per gonad arm of nematodes after 24, 48, and 72 h of exposure; (**D**) comparison of effects of TP on the number of oocytes in diakinesis per gonad arm of nematodes after 24, 48, and 72 h of exposure; (**E**) effects of TP on the morphology of DTCs after 24 h of exposure (scale bar, 50 µm); (**F**) effects of TP on mitotic germ cells after 24 h of exposure, where the left area beside the white line contains mitotic cells (scale bar, 10 µm); (**G**) effects of TP on apoptosis after 24 h of exposure, where the arrow indicates apoptotic cells (scale bar, 50 µm); and (**H**) effects of TP on oocytes in diakinesis after 24 h of exposure (scale bar, 50 µm). TP, Triptolide. Bars represent means ± SEM. * *p* < 0.05 vs. the control group.

In terms of the relative fluorescence intensity of DTCs, there was no significant difference between the control and the Triptolide-exposed nematodes (Figure S1). However, at 24 and 48 h, the area of DTCs was altered, and significantly shrunk in nematodes that were exposed to 50 and 100 mg/L Triptolide for 4 h (Figure 8A). Figure 8E shows the images taken at 24 h. Similarly, at 72 h after exposure, the area of DTCs was significantly shrunk in nematodes that were exposed to 100 mg/L Triptolide.

### 2.7. Effects of Triptolide on Mitotic Germ Cell Proliferation

After 24 and 48 h of exposure, the number of mitotic germ cells per gonad arm was significantly decreased in nematodes that were exposed to 50 and 100 mg/L Triptolide. Similarly, after 72 h of exposure, the number of mitotic germ cells per gonad arm was significantly decreased in nematodes that were exposed to 100 mg/L Triptolide (Figure 8B). Figure 8F shows the images taken at 24 h.

### 2.8. Effects of Triptolide on Apoptosis

After 24 and 48 h of exposure, the number of apoptotic cells per gonad arm was significantly increased in nematodes that were exposed 50 and 100 mg/L of Triptolide. After 72 h of exposure, the number of apoptotic cells per gonad arm was significantly increased only in nematodes that were exposed to 100 mg/L Triptolide (Figure 8C). Figure 8G shows the images taken at 24 h.

### 2.9. Effects of Triptolide on Oocytes in Diakinesis Stage

As shown in Figure 8D, after 24 and 48 h of exposure, there was a significant decrease in the number of oocytes in diakinesis stage per gonad arm in nematodes that were exposed to 50 and 100 mg/L Triptolide. After 72 h of exposure, the number of oocytes in diakinesis stage per gonad arm was significantly reduced in nematodes that were exposed to 100 mg/L Triptolide. Figure 8H shows the images taken at 24 h.

**Figure 9:**
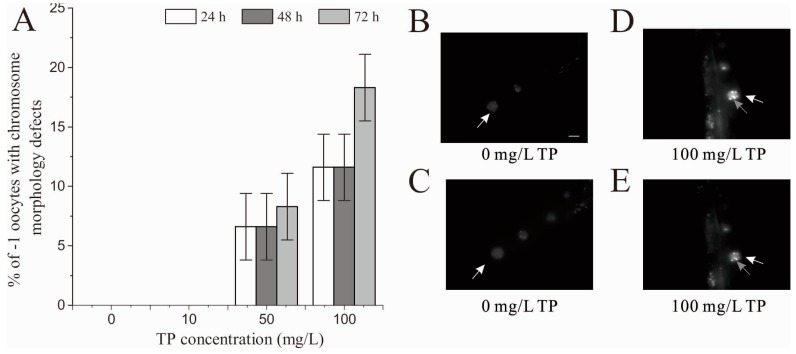
Effects of exposure to TP for 4 h on the chromosome of −1 oocytes in diakinesis: (**A**) percent of −1 oocytes with chromosome morphology defects; (**B**,**C**) the chromosome of the same −1 oocyte in diakinesis of the control group on different focal planes, with six intact DAPI-stained bodies distributed dispersedly in the nucleus; and (**D**,**E**) the chromosome of the same −1 oocyte in diakinesis of 100 mg/L TP group on different focal planes. A chromatin aggregate was observed in the nucleus. White arrows point to the −1 oocytes. Grey arrows point to the chromatin aggregates. Scale bar is 10 µm. TP, Triptolide. Bars represent means ± SEM.

As shown in Figure 9A, after 24, 48 and 72 h of exposure, there was no chromosome morphology defects observed in −1 oocytes of nematodes exposed to 10 mg/L Triptolide. Chromosome morphology defects were observed in −1 oocytes of nematodes exposed to 50 and 100 mg/L Triptolide. Figure 9B–E shows the images of the control and nematodes exposed to 100 mg/L Triptolide taken at 24 h. The chromosome morphology defects mainly include chromatin aggregates.

### 2.10. Effects of Triptolide on the Expression Patterns of Genes Involved in the Regulation of Oocyte Properties

According to the results described in the previous sections, oocytes at different developmental stages had the most significant changes after 24 h of the 4 h exposure to Triptolide. Thus, we analyzed the expression patterns of various genes involved in the regulation of oocyte properties after 24 h of exposure.

**Figure 10:**
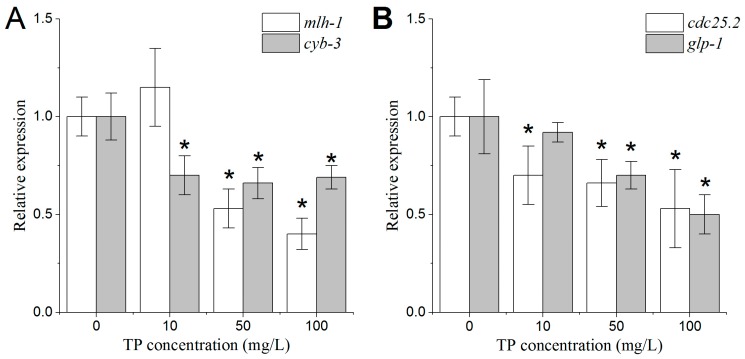

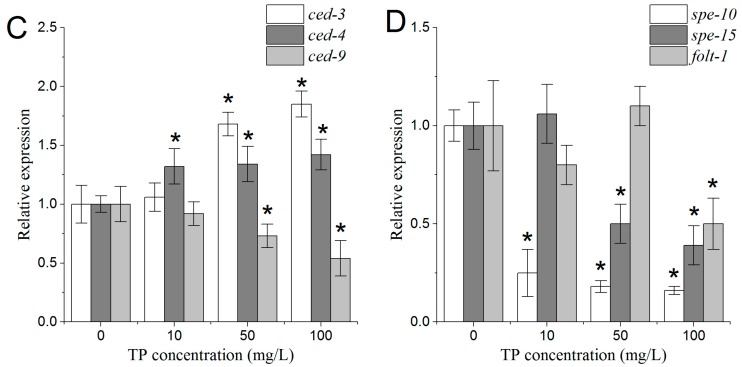
Relative mRNA expression levels of genes involved in gametogenesis of L4 larval nematodes exposed to TP. Gene expression was analyzed at 24 h after the exposure: (**A**,**B**) relative mRNA expression levels of genes involved in mitotic germ cell proliferation and oocyte quality; (**C**) relative mRNA expression levels of genes involved in apoptosis; and (**D**) relative mRNA expression levels of genes involved in spermatogenesis. TP, Triptolide. Bars represent means ± SEM. * *p* < 0.05 vs. the control group.

As shown in Figure 10A,B, the expression levels of *mlh-1* and *glp-1* were significantly decreased in nematodes that were exposed to 50 and 100 mg/L Triptolide. Moreover, the expression levels of *cdc-25.2* and *cyb-3* were significantly reduced in nematodes that were exposed to all concentrations of Triptolide.

The expression levels of *ced-3* and *ced-4* were increased in nematodes that were exposed to 50 and 100 mg/L Triptolide. In contrast, the expression level of *ced-9* was decreased in nematodes that were exposed to 50 and 100 mg/L Triptolide (Figure 10C).

### 2.11. Effects of Triptolide on the Expression Patterns of Genes Involved in the Regulation of Spermatids Properties

After 24 h of exposure to Triptolide, the genes involved in the regulation of spermatids properties were measured. As shown in Figure 10D, the expression level of *spe-10* gene was significantly decreased in nematodes that were exposed to all concentrations of Triptolide. Moreover, the expression level of *spe-15* was significantly reduced in nematodes that were exposed to 50 and 100 mg/L Triptolide. The expression level of *folt-1* was significantly reduced in nematodes that were exposed to 100 mg/L Triptolide.

## 3. Discussion

*Tripterygium wilfordii* glycosides are widely used in clinical practice, to treat autoimmune diseases, dermatitis, and other illnesses. Clinical observations indicated that the incidence of side effects of *Tripterygium wilfordii* glycosides was higher in women than in men, and that damage to the reproductive system was one of the most serious side effects. The main clinical side effects of treating female patients with *Tripterygium wilfordii* glycosides are menstrual disorders, including irregular menstruation and amenorrhea. Among these symptoms, amenorrhea has the highest incidence (14.2%) [22].

Triptolide is the most effective ingredient of *Tripterygium wilfordii* glycosides after their separation. It was reported that Triptolide can induce damage to the ovaries, decrease the number of oocytes, and inhibit the proliferation of granulosa cells [23,24]. The elucidation of the mechanisms of the effects of Triptolide requires further investigations, in order to better guide the clinical application of *Tripterygium wilfordii* glycosides.

It is well known that decreased ovarian function is one of the main causes of amenorrhea. In studies dealing with toxicity to the female reproductive system, the fact that the oocyte is not readily available for study constitutes an experimental bottleneck. The method of in vitro follicle culture has certain limitations in the field of risk assessment because of the lack of normal physiological conditions. Alternative model organisms, such as *C. elegans*, constitute a bridge between in vitro testing methods and rodent testing for risk assessment. *C. elegans* has been regarded as a powerful model for assessing the chemical disruption of germline functions [25,26]. An advantage of *C. elegans* as a model organism is that the effects of exogenous chemicals on the oocytes at different developmental stages can be easily observed. We used *C. elegans* to explore the effects of Triptolide on the germline of *C. elegans* and to elucidate possible pharmacological mechanisms involved. We aimed to provide a basis for the scientific use of the drug in clinical practice.

We found that 4 h of exposure to 50 and 100 mg/L Triptolide significantly reduced *C. elegans* fertility, indicating that Triptolide exerts its toxic effects on the germline. Spermatogenesis begins at L4 larvae. To distinguish whether induced sperm or oocyte gives rise to the decrease in brood size, we first analyzed the morphological development and function of spermatids, and the expression levels of related genes. The result showed that the number of spermatids, the size of spermatids, and the rate of spermatids activation were significantly changed after the Triptolide exposure. The genes involved in the above properties were selected to study the potential damaging mechanisms. The *C. elegans* gene *folt-1* is an ortholog of the human reduced folate carrier gene. FOLT-1 is related with nematodes fertility and sperm count [27,28]. SPE-10 is required for the spermatids morphogenesis and motility function, and its loss-of-function mutants have smaller spermatids compared with the normal ones [29,30]. *Spe-15* is a key gene in spermiogenesis [31]. The change in *folt-1* gene expression level was consistent with the change in the number of spermatids, which indicates that Triptolide might inhibit the spermiogenesis by disturbing the utility of folate. The changes in *spe-10* and *spe-15* genes expression levels corresponded with the changes in size of spermatids and the spermatids activation, which indicates that Triptolide might damage the morphogenesis and motility function of spermatids.

To study the effects of Triptolide on oocytes, the mating experiments with Triptolide-exposed wild type hermaphrodites and *him-5* males were conducted. It was shown that the number of unfertilized oocytes of crosses between *him-5* males and wild type hermaphrodites exposed to 50 and 100 mg/L of Triptolide was significantly increased, which indicates that Triptolide led to the decrease in oocyte quality. Alterations in the mechanisms of sister chromatid segregation and DNA damage repair are most likely the factors leading to the decrease in oocyte quality. In the previous studies, it was found that MLH-1, CYB-3, and CDC-25.2 were involved in the processes of mitosis and DNA damage repair. We therefore selected the genes *mlh-1*, *cyb-3*, and *cdc-25.2* to study the oogenesis damaging mechanisms of Triptolide. The *mlh-1* gene belongs to the DNA mismatch repair gene; it encodes a DNA mismatch repair protein homolog that is orthologous to human MLH-1. CYB-3 encodes one of the four *C. elegans* cyclin B family members that are most closely related to B3-type cyclins. CYB-3 is required for a number of dynein-related mitotic processes, specifically for the initiation of chromosome segregation during anaphase. RNAi to block *cyb-3* or *mlh-1* increases the percentage of unfertilized oocytes [32]. CDC-25.2 is orthologous to human CDC25, and is required to promote oocyte maturation. *Cdc-25.2* mutant oocytes were arrested as endomitotic oocytes that were not fertilized successfully [33]. Moreover, decreased expression levels of *mlh-1*, *cyb-3*, and *cdc-25.2* were consistent with an increase in the number of unfertilized oocytes in the present study. This indicates that Triptolide might adversely affect oocyte quality by damaging chromosomes and by altering DNA damage repair mechanisms.

To further study the toxic effects of Triptolide on the oocyte development and the possible mechanisms involved, we analyzed the morphological changes of DTCs, the changes of the number of mitotic germ cells, the changes of the number of apoptotic cells, and the changes of the oocytes in diakinesis stage. To perform this analysis we used transgenic nematode strains with specific markers: JK2868 strain for the analysis of DTCs, and OD73 strain for the analysis of oocytes. Using these transgenic strains, the above endpoints are quantitative and easily measurable, among which the area of DTCs is firstly used in the risk assessment of chemicals on the reproductive system. Our results showed that, after 24 and 48 h of the 4 h exposure to 50 and 100 mg/L Triptolide, the DTCs had shrunk, that the number of mitotic germ cells and oocytes in diakinesis stage per gonad arm was reduced, and that the number of apoptotic cells per gonad arm was augmented. The DTCs is critical for proliferation of the germline. The DTCs control germline proliferation during larval development and control germline mitoses in adults. In the germline of *C. elegans*, GLP-1 is essential for mitotic proliferation of germ cells. The decreases in the expressive levels of *glp-1* in nematodes exposed to 50 and 100 mg/L corresponded with the decrease in the number of mitotic germ cells. The shrunken shape of DTCs and the decreased number of mitotic germ cells evidenced the adverse effects of Triptolide on the proliferation of the germline.

In *C. elegans*, apoptosis of the germline occurs only during oocyte production, and therefore is only observed in adult hermaphrodites. Apoptosis of the germline is restricted to the gonad loop region, where developing oocytes exit at the pachytene stage of meiotic prophase and transition into diplotene. Previous studies have shown that the exposure to certain environmental stressors, and to specific chemicals, could increase the number of germ cells in apoptosis [34,35,36,37]. The present study also showed that exposure to 50 and 100 mg/L Triptolide for 4 h significantly increased the number of germ cells in apoptosis, indicating that Triptolide affects the development of oocytes through the inhibition of oocyte transition from pachytene to diplotene. In addition, it has been reported that, as the rate of germ cell proliferation declines markedly in aging hermaphrodites, the continuation of physiological apoptosis becomes a pathogenic mechanism that promotes gonad degeneration [38]. Based on the changes in proliferation and apoptosis in nematodes that were exposed to 50 and 100 mg/L Triptolide, we predict that gonad senescence could be accelerated, leading to the final decline of ovarian function. Physiological germ cell apoptosis of *C. elegans* occurs in the absence of exogenous stressors and is initiated by the core apoptotic pathway. In the core apoptotic pathway, the genes *ced-3* and *ced-4* promote the apoptosis, while *ced-9* inhibits it [39]. In addition, it is evident that multiple noncanonical genetic pathways lead to the apoptosis of germ cells, including DNA-damage checkpoint pathway, meiotic recombination pathway, and MAP kinase pathway [40,41,42]. Until now, there have been few studies on the genetic toxicity of Triptolide. Thus, we selected the core apoptotic pathway preferentially to study the mechanisms. The present study found that the expression levels of *ced-3* and *ced-4* were augmented and that the expression level of *ced-9* was diminished, further supporting the observation that Triptolide increased the number of apoptotic cells in the gonad arms of nematodes after 24 h of exposure. These findings suggest that the potential to form mature oocytes was suppressed in Triptolide-exposed nematodes.

It is documented that the apoptosis of germ cells is regulated by multiple genetic pathways. Some previous studies found that some chemicals induced the increased apoptosis of germ cells through DNA-damage checkpoint pathway [36,37], indicating that other pathways might also be involved in the process of increased apoptosis induced by Triptolide. Furthermore, Triptolide might be genotoxic in a certain dose range because the chromatin aggregation was observed in -1 oocytes of nematodes exposed to 100 mg/L Triptolide.

After the pachytene stage of meiosis, oocytes enter into diakinesis and reach the endpoint of development. The present study found a decrease in the number of oocytes per gonad arm (from the spermatheca to the loop region), which means that Triptolide impaired the development of oocytes in diakinesis.

Previous studies have shown that Triptolide might cause a decline of fertility in male rats. However, after the exposure to Triptolide stopped, male rat fertility was restored to some extent [43,44]. It is still unknown whether the toxic effects of Triptolide on the fertility of female rats are reversible. The present study has shown that brood size in nematodes that were exposed to 50 and 100 mg/L Triptolide was significantly decreased in the first, second and third days of the spawning time. However, the difference in brood size between the control nematodes and nematodes that were exposed to 100 mg/L Triptolide in the first spawning day was most obvious. In addition, after 72 h of exposure to 50 mg/L Triptolide, the shape of DTCs, the number of mitotic cells, the number of apoptotic cells, and the number of oocytes were not significantly different from the controls, which indicates that the damage of Triptolide to the germline of *C. elegans* is present even after Triptolide exposure has stopped, but the damage induced by Triptolide at the 50 mg/L dose to germline is reversible.

## 4. Materials and Methods

### 4.1. Chemicals

Triptolide (CAS#: 38748-32-2, No. 111567-200603, purification ≥ 98%) was purchased from Jiangsu Institute for Food and Drug Control of China (Nanjing, China), and it was produced by the National Institutes for Food and Drug Control. One milligram Triptolide was dissolved in 50.0 µL DMSO, ultrasound bathed at 50 °C for 30 min, shaken well, and finally progressively diluted with K medium (0.032 M KCl, 0.051 M NaCl) to 10, 50, and 100 mg/L dosing solutions of Triptolide [45]. The pH of dosing solution was 7.8. Pronase was obtained from Roche (Basel, Switzerland), and other chemicals were obtained from Sigma-Aldrich (St. Louis, MO, USA).

### 4.2. Strain Preparation

Nematodes used for the present study were wild-type N2, *him-5* (*e1490*), JK2868 (*qIs56v*), and OD73 (*unc-119*(*ed3*) *III*; *ItIs38*; *ItIs24*). These nematode strains have specific markers that are important for our microscopic analysis: JK2868 stain has markers for DTCs, and OD73 strain has markers for the cytomembrane of oocytes. These strains were originally obtained from *Caenorhabditis* Genetics Center, USA. They were maintained on nematode growth media (NGM) plates seeded with *Escherichia coli* OP50 at 20 °C as described by Brenner (1974) [46]. Synchronous age populations of L4 larvae nematodes were obtained by transferring two healthy adult nematodes to a fresh NGM plate, and picking L4 larvae nematodes after 3 days. Exposure to Triptolide was performed in 12-well sterile tissue culture plates at 20 °C in the presence of food.

### 4.3. Lethal Toxicity and Measurement of Body Length

For lethal toxicity assays, a 1.0 mL aliquot of the test solutions (10, 50, or 100 mg/L Triptolide) was added to each well of tissue culture plates. Wells were then loaded with L4 larvae of N2 nematodes for each treatment. After 4 and 8 h of exposures, wells were observed under a dissecting microscope, the number of dead nematodes was scored. Fifty nematodes were used and three replicates were examined per treatment.

At 24 h after L4 larvae of N2 nematodes were exposed to Triptolide for 4 and 8 h, body length was determined by measuring the flat surface area of adult nematodes along the anterior-posterior axis using Image-Pro^®^ Express 6.0 software (Media Cybernetics, Rockville, MD, USA). Twenty nematodes were used and three replicates were performed per treatment.

### 4.4. Reproduction Assays

The analysis of brood size of N2 nematodes was performed as described previously [47]. L4 larvae of N2 nematodes were exposed to Triptolide for 4 h. To analyze brood size, the number of offspring at all developmental stages and the number of eggs were scored under a dissecting microscope. Ten nematodes were used and three replicates were performed per treatment.

### 4.5. Number of Unfertilized Oocytes

After L4 larvae of N2 nematodes were exposed to Triptolide for 4 h, one exposed nematode was placed with a *him-5* male for mating, and then the N2 nematodes were transferred to new plates every 12 h until egg production ceased. The number of unfertilized oocytes was counted. Unfertilized oocytes do not form egg shells. They are oval with a light color. However, nonviable eggs form egg shells with sharp edges and darker than unfertilized oocytes. Ten nematodes were used and three replicates were performed per treatment.

### 4.6. Number of Spermatids

After L4 larvae of N2 nematodes were exposed to Triptolide for 4 h, the exposed nematodes were placed to a NGM plate with food for 6 h. Then, they were put into a drop of sperm medium solution (50 mM Hepes, 1 mM MgSO_4_, 25 mM KCl, 45 mM NaCl, 5 mM CaCl_2_, and pH 7.0) with polyvinyl pyrrolidone on a microscope slide [48]. Spermatids were dissected from hermaphrodites by cutting at the neighboring zone of spermatheca. The number of spermatids was scored under a microscope (Zeiss Scope A1, Zeiss, Oberkochen, Germany). Ten nematodes were used and three replicates were performed per treatment.

### 4.7. Morphology of Spermatids

The sperm size and shape were measured and analyzed according to a previous description [18]. After L4 larvae of N2 nematodes were exposed to Triptolide for 4 h, the exposed nematodes were placed to a NGM plate with food for 6 h. The spermatids were acquired the same way as Section 4.6. Five different visual fields for each sample were captured under a microscope (Zeiss Scope A1, Zeiss) randomly, and images were acquired with a charge coupled device (CCD) camera. The shape of spermatids was observed, and the diameter of spermatids was measured from CCD-captured images using the ZEN pro 2011sp 6.0 software (Zeiss).

### 4.8. Rate of Spermatids Activation In Vitro

The spermatids activation was performed according to the description [49]. L4 larvae of N2 nematodes were exposed to Triptolide for 4 h, and the exposed nematodes were placed to a NGM plate with food for 6 h. After spermatids were dissected in a small drop of sperm medium solution, a drop of 200 μg/mL pronase was added to the sperm medium solution. After 5 min treatment, the number of sperm with a pseudopod was counted under a Zeiss Scope A1 microscope (Zeiss). The rate of spermatids activation was calculated by the number of sperm with a pseudopod, divided by the total number of spermatids. Ten nematodes were used and three replicates were performed per treatment.

### 4.9. DTC Developmental Assays

L4 larvae of JK2868 strain were exposed to Triptolide for 4 h. Then, pictures of DTCs were taken under a fluorescence microscope (Zeiss Scope A1) at 24, 48 and 72 h after the exposure. The relative fluorescent intensities of DTCs were semi-quantified using Image-Pro^®^ Express 6.0 software (Media Cybernetics). The area of the umbrella-shaped cell body of DTCs was measured using the ZEN pro 2011 Hardware (Zeiss). A normal DTC is light green and umbrella-shaped. Ten nematodes were used and three replicates were performed per treatment.

### 4.10. Mitotic Germ Cell Proliferation Arrest Assays

Mitotic germ cell proliferation arrest was assessed as described previously [50]. In the germline of hermaphrodite, the germ cells in the transitional zone can be easily identified due to the crescents of DNA. To count the number of mitotic germ cells, the start of the transitional zone was defined as the column when crescents accounted for more than 30% of the total cells at the distal end. Then, the number of mitotic germ cells was counted between the DTC and the start of the transitional zone. L4 larvae of N2 nematodes were exposed to Triptolide for 4 h. Then, the larvae were transferred to a new NGM and aged for about 22, 46 and 70 h. Adult nematodes were then stained with DAPI. The mitotic nuclei at the distal end of the germline were counted blind using a Zeiss Scope A1 fluorescence microscope (Zeiss). Ten nematodes were used and three replicates were performed per treatment.

### 4.11. Germ Cell Apoptosis Assays

Apoptotic cells in germline were stained as described previously [51]. L4 larvae of N2 nematodes were exposed to Triptolide for 4 h. Then, the larvae were transferred to a new NGM and aged for about 22, 46 and 70 h. Adult nematodes were picked into 100 µL 25 µg/mL AO in M9 in 1.5 mL tubes and stained for 2 h in a 20 °C incubator. Some OP50 were added to the AO tube facilitating uptake of the dye. Nematodes were allowed to recover for 10 min on a new NGM plate and then mounted in an agar pad. The number of apoptotic cells per gonad arm was scored using a Zeiss Scope A1 fluorescence microscope (Zeiss). Ten nematodes were used and three replicates were performed per treatment.

### 4.12. Number of Oocytes Per Gonad Arm and Chromosome Morphology of −1 Oocytes in Diakinesis Stage

L4 larvae of OD73 strain were exposed to Triptolide for 4 h. Then, the number of oocytes located in each gonad arm (on the area from the spermatheca to the gonad loop) was scored at 24, 48 and 72 h after the exposure, using a Zeiss Scope A1 fluorescence microscope (Zeiss). Ten nematodes were used and three replicates were performed per treatment.

L4 larvae of N2 nematodes were exposed to Triptolide for 4 h. Chromosome of oocytes in diakinesis was stained with DAPI as the previous study [52]. Chromosome morphology of −1 oocytes in diakinesis was assessed as described previously [25]. At 24, 48 and 72 h after the exposure, the nematodes were fixed by 4% paraformaldehyde, stained with 0.2 µg/mL DAPI for 30 min, washed by PBSB three times, and then mounted for observation on an agar pad. The chromosome in −1 oocytes (the first oocyte next to the spermatheca) was observed using a Zeiss Scope A1 fluorescence microscope (Zeiss). Every −1 oocyte was observed on different focal planes and their photos taken to assess the chromosome. Twenty nematodes were used and three replicates were performed per treatment.

### 4.13. Real-Time qRT-PCR

We selected all the genes through reading relevant references [15,16,27,28,29,30,31,32,33,39] and abiding by the principle of homolog. Then, they were confirmed using the public network “wormbase” (available online: www.wormbase.org).

The procedure of real-time qRT-PCR is as follows. Approximately 6000 L4 larvae of N2 nematodes were exposed to different concentrations of Triptolide for 4 h. Total RNA of each group was extracted 24 h later using RNeasy Mini Kit (Qiagen, Suzhou, China). The quantity and quality of RNA were analyzed with a Nanodrop 1000 Spectrophotometer (Thermo Fisher Scientific, Wilmington, DE, USA). Total high-quality RNA was used for cDNA synthesis using PrimeScript^TM^RT Master Mix (Takara Bio, Dalian, China). Real-time PCR was performed on the cDNA products using SYBR Green I as a dye in a LightCycler^®^ 96 Real-Time PCR System (Roche, Basel, Switzerland). The primers tested are shown in Table 1.

The mRNA levels were analyzed using the comparative *C*_t_ method. For normalization of individual samples, the initial level of target cDNA was expressed as the difference in *C*_t_ values (Δ*C*_t_) between the target and an internal control *act-1*, which is a constitutively expressed gene. Differences in gene expression were calculated using the comparative *C*_t_ method [53]. A dissociation curve analysis was obtained after the final PCR cycle to evaluate the presence of nonspecific PCR products and primer dimers. Three replicates were conducted for each analysis.

### 4.14. Statistical Analysis

All data in this study are expressed as mean ± standard error of the mean (SEM). Statistical analyses were performed using SPSS 12.0 (SPSS Inc., Chicago, IL, USA). Differences between groups were determined using an analysis of variance (ANOVA). Probability levels of 0.05 were considered statistically significant.

## 5. Conclusions

Triptolide severely damaged the fertility of *C. elegans*, impeded oogenesis, and reduced oocyte quality. These effects were related to damage to the morphogenesis of DTCs, decreased number of mitotic germ cells, increased number of germ cells in apoptosis, decreased number of oocytes in diakinesis stage, and chromatin aggregates in −1 oocytes. We have identified genes involved in the damaging effects of Triptolide on *C. elegans* oogenesis and fertility. Importantly, *C. elegans* was sensitive to Triptolide, which is a known reproductive toxicant of mammals. We thus recommend the use of this alternative model for assessing how chemicals damage fertility through the damage of oogenesis mechanisms.

## Figures and Tables

**Table 1:** Gene primers tested in the study.

Gene Primer	Sequence	Annealing Temperature
*mlh-1*	Forward GAGAAGACGATGATGTGGA	50.3 °C
	Reverse GAGATGGTAGAGGGAGGTG	
*cyb-3*	Forward ATGGTCTCACAATCCCGTC	62.9 °C
	Reverse GTGGCATCACCTCCTCTCC	
*cdc-25.2*	Forward GCCAGGTGCGTCTGTTCG	54.2 °C
	Reverse CGTTTCCGCTGCTGTAGG	
*glp-1*	Forward AACTGTTGTCGCTGGTGT	50.0 °C
	Reverse TCTCTTGGTATTGGGGTG	
*ced-3*	Forward ACGGGAGATCGTGAAAGC	53.0 °C
	Reverse AGAGTTGGCGGATGAAGG	
*ced-4*	Forward AGTCACTCGCAATGGCTCT	57.5 °C
	Reverse GCTGATGAACGACGGAAT	
*ced-9*	Forward AAAGGCACAGAGCCCACC	50.0 °C
	Reverse CGTTCCCATAACTCGCATC	
*folt-1*	Forward TCCATTCCTCACTCCGTTTCTA	60.4 °C
	Reverse GCATCTGCCATACTCCTTTACC	
*spe-10*	Forward TTTTATTGTCGGCGGAGTGT	57.8 °C
	Reverse CGATGACTGCGAACTTTGAG	
*spe-15*	Forward GGAGTTTTGGATGTCGCTGGTT	60.4 °C
	Reverse GCTCTCTGGGTGAAATGTTGGA	
*act-1*	Forward ATGTGTGACGACGAGGTT	60.4 °C
	Reverse GAAGCACTTGCGGTGAAC	

## Supplementary Materials

Supplementary materials can be found at www.mdpi.com/1422-0067/18/2/464/s1.

## Abbreviations

*Caenorhabditis elegans*	*C. elegans*
TP	Triptolide
DTC	Distal tip cell
DAPI	4,6-diamidino-2-phenylindole
