# Supplementary material for: The Adverse Effects of Triptolide on the Reproductive System of Caenorhabditis elegans: Oogenesis Impairment and Decreased Oocyte Quality
Source: Int J Mol Sci. 2017 Feb 21;18(2):464. doi: 10.3390/ijms18020464 (PMC5343997; doi:10.3390/ijms18020464)
Supplement: Supplementary file 1 [file ijms-18-00464-s001.pdf]

Supplementary Materials: The Adverse Effects of Triptolide on the Reproductive System of *Caenorhabditis elegans*: Oogenesis Impairment and Decreased Oocyte Quality

Qinli Ruan, Yun Xu, Rui Xu, Jiaying Wang, Yongqing Hua, Meng Wang and Jinao Duan

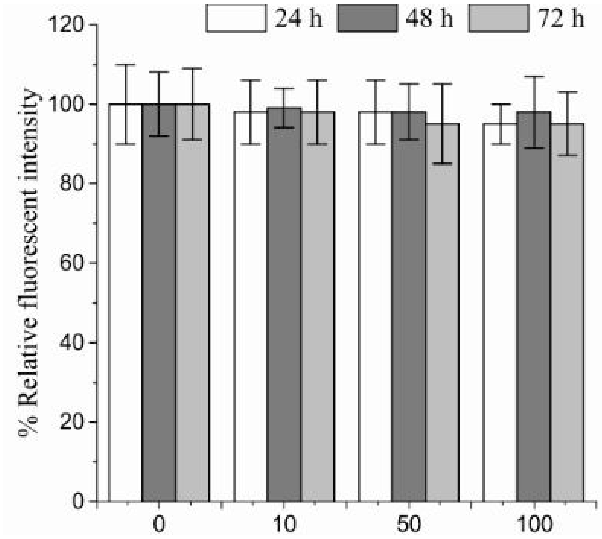

Figure S1. Comparison of the effects of Triptolide on the relative fluorescent intensity of the DTCs after 24, 48, and 72 h of the exposure.
